# Interobserver variability in high-resolution CT of the lungs

**DOI:** 10.1016/j.ejro.2020.100228

**Published:** 2020-03-31

**Authors:** Jonas Widell, Mats Lidén

**Affiliations:** Department of Radiology, Örebro University Hospital, Region Örebro County, Sweden

**Keywords:** HRCT, High-resolution Computed Tomography, MDCT, Multi-detector Computed Tomography, ATS/ERS/JRS/ALAT, American Thoracic Society/European Respiratory Society/Japanese Respiratory Society/Latin American Thoracic Society, IPF, Idiopathic Pulmonary Fibrosis, UIP, Usual Interstitial Pneumonia, CI, Confidence Interval, HRCT, Usual interstitial pneumonia, Idiopathic pulmonary fibrosis, Interobserver variation, Inter-reader variation

## Abstract

**Purpose:**

To quantify the interobserver variability among the most frequently encountered parenchymal patterns in High Resolution CT (HRCT) and to compare the interobserver variability in the application of the 2011 and 2018 usual interstitial pneumonia (UIP) criteria according to the joint guidelines from international thoracic and respiratory societies.

**Material and methods:**

Two observers independently evaluated 126 HRCT, with examples of most common parenchymal patterns, and noted the presence of each pattern. The readers also noted whether the findings met the 2011 criteria for UIP. In a second reading, the same readers noted whether the HRCT met the UIP criteria according to the 2018 UIP update.

**Results:**

The kappa values for interobserver variability for the different patterns ranged from 0.28 (intralobular lines) to 0.85 (tree-in-bud nodules). The kappa value for UIP pattern was similar for 2011 and 2018 criteria, 0.58 and 0.69, respectively. Compared to the 2011 UIP criteria, there was no statistically significant difference in the number of HRCT classified as UIP using the 2018 criteria.

**Conclusions:**

There is a substantial variation in interobserver agreement between the different parenchymal patterns, which suggests that some patterns a more easily identified than others. There is also a considerable reader variation in the assessment of UIP applying the 2011 UIP criteria as well as applying the 2018 UIP update.

## Highlights

• There are substantial differences in interobserver agreement between different patterns in HRCT.
• The highest agreement was seen for tree-in-bud nodules, and the lowest agreement was seen for intralobular lines.
• Although the agreement for honeycombing was high, the agreement for the UIP criteria was considerably lower.

## 1 Introduction

High-resolution computed tomography (HRCT) of the lungs is the best non-invasive method to assess the lung parenchyma [1]. Even subtle changes in the lung tissue can be demonstrated in the HRCT images thanks to the thin slices and high spatial frequency reconstruction algorithm. Since its introduction in the 1980´s, the examination technique has continuously evolved. Nowadays, multi-detector computed tomography (MDCT) enables continuous thin slices and multiplanar reconstructions [2,3].

The publication of the ATS/ERS/JRS/ALAT guideline for diagnosis of idiopathic pulmonary fibrosis (IPF) in 2011 emphasized the importance of HRCT in interstitial lung disease (ILD) [4]. The identification of a typical HRCT appearance is sometimes sufficient to provide a certain diagnosis. In the appropriate clinical context, if the HRCT findings meet the criteria for Usual Interstitial Pneumonia (UIP), IPF can be confidently diagnosed, obviating the need for a surgical lung biopsy [[4], [5], [6]].

The 2011 guideline with its HRCT criteria for “UIP Pattern”, “Possible UIP Pattern” and “Inconsistent with UIP Pattern”, was an important milestone for standardizing the assessment of IPF using HRCT [4]. The ATS/ERS/JRS/ALAT guideline was updated in 2018. In the 2018 UIP criteria, the HRCT is classified into four categories; “UIP”, “Probable UIP”, “Indeterminate for UIP” and “Alternative Diagnosis” [6].

Interpretation of HRCT – in IPF and other ILD – relies on the identification of typical parenchymal patterns and their distribution within the lungs [[6], [7], [8]]. However, most patterns are unspecific, there is an overlap in the radiological appearance between different diseases, and the same disease may show many different appearances. This complexity underlines the importance of the multi-disciplinary collaboration for correct interpretation of HRCT in IPF, but also in other ILD [6,9].

From the radiologists' perspective, a consistent assessment of the typical HRCT patterns is crucial for accurate interpretation of HRCT. Consequently, several studies have investigated the intra- and interobserver variations in HRCT. Previous studies have focused on specific lung diseases and specific patterns, for example the 2011 UIP criteria, interstitial lung diseases, bronchiectasis and asbestos related changes [[10], [11], [12], [13], [14], [15], [16], [17]]. However, to the best of our knowledge, there is no study that has simultaneously addressed the interobserver variability for the wide range of typical HRCT patterns. The difference between specific patterns in interobserver variability remains unknown.

The purpose of the present study was, therefore, to quantify the interobserver variability among the most frequently encountered parenchymal patterns in HRCT, and to compare the interobserver variability in the application of the 2011 and 2018 UIP criteria.

## 2 Material and methods

The study was performed in three phases. The first phase was the creation of an HRCT image databank including several examples each from a predefined list of typical parenchymal patterns. Subsequently, two readings of the databank were performed to assess the interobserver variability for the parenchymal patterns and for the 2011 and 2018 UIP criteria.

The regional research ethics board approved the study protocol and waived the informed consent requirement.

### 2.1 HRCT image databank

Because of the uneven distribution of parenchymal patterns in any clinical cohort, a specially created databank is necessary for the analysis of interobserver variability of the typical HRCT patterns. An anonymous HRCT databank was created for the study, consisting of 126 HRCT examinations with several examples of each of the typical patterns and also examples of examinations demonstrating no pathological parenchymal patterns. The inclusion in the databank followed a predefined list of patterns to be included.

The inclusion criterion was continuous slice HRCT examination demonstrating any of the predefined patterns. The patterns that were included in the databank were perilymphatic nodules, tree-in-bud nodules, other centrilobular nodules, ground glass opacicties, thickened interlobular septa, intralobular lines, septations and lines, consolidation with and without air bronchogram, crazy paving, emphysema, honey combing and other cystic patterns. Exclusion criteria were contrast enhanced examination and considerable artifacts, for example artifacts from respiratory motion or metal implants.

**Fig. 1:**
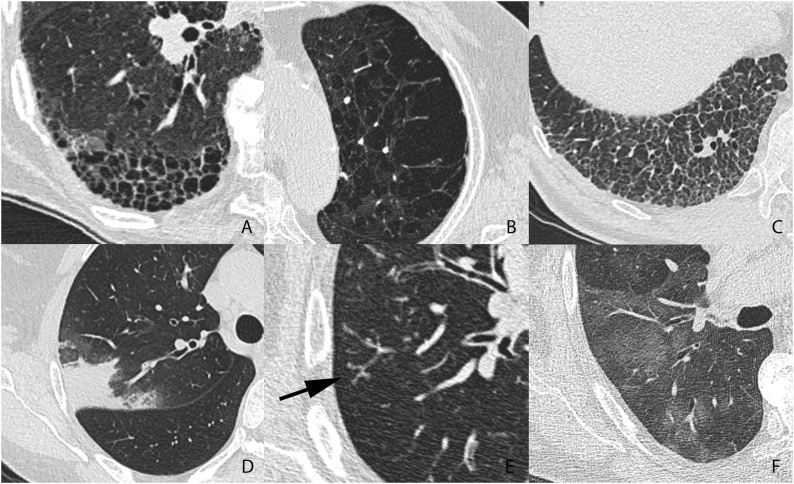
Examples of parenchymal patterns. A. Honeycombing. B. Emphysema. C. Reticular pattern. D. Consolidation. E. Tree-in-bud (arrow). F. Ground glass.

The inclusion of examinations with each pattern was discontinued when the predefined number of examinations demonstrating the specific pattern was obtained. Examples of patterns included in the databank are shown in Fig. 1.

One observer (with 4 years of experience of thoracic imaging) performed the inclusion in the databank. The databank was retrospectively created by reviewing all HRCT examinations performed during 39 randomly selected months between 2011 and 2016 in the region of Örebro, Sweden, which consists of one university hospital and two smaller associated hospitals. Inclusion during randomly selected months was a part of the anonymization process.

A continuous slice HRCT was defined as a thoracic scan in supine position with breath hold at full inspiration with continuous ≤ 1 mm images, reconstructed using a sharp kernel.

All examinations were of normal radiation dose (ref-mAs ∼150, ref-kV 120). The CTDI was not available in the anomymized databank. The studies were acquired with Philips Ingenuity CT (n = 4), Ingenuity Core 128 (n = 13), Brilliance 16 (n = 1), Brilliance 40 (n = 7) or with Siemens Biograph 40 (n = 12), Somatom Definition AS (n = 55), Somatom Definition AS+ (n = 2) and Somatom Definition Flash (n = 32). Reconstruction algorithms were L (n = 25), B70f (n = 68), I70f\1 (n = 20), I70f\2 (n = 11) or I70f\3 (n = 2). The images were acquired at 100 kVp (n = 20), 120 kVp (n = 97) or 140 kVp (n = 9).

### 2.2 Image analysis

Two readers (radiologists with 4 and 6 years of experience of thoracic imaging) independently evaluated the 126 HRCT in the databank in two separate readings. In the first session, the readers noted all identifiable patterns in each HRCT, using a score sheet with the same list of patterns as for the creation of the databank. The readers also assessed whether the HRCT findings met the criteria for “UIP Pattern” according to the 2011 ATS/ERS/JRS/ALAT criteria [4]. Scans classified as “Possible UIP Pattern“or “Inconsistent with UIP Pattern” were not separated.

In a second reading, separated by more than one year from the first reading, the readers classified the HRCTs according to the 2018 UIP criteria update [6]. In this classification, each HRCT was classified in one of the four classes “UIP”, “Probable UIP”, “Indeterminate for UIP” or “Alternative Diagnosis”.

The first observer also created the databank. The creation of the databank was separated from the first reading with at least three months.

### 2.3 Statistical analysis

The interobserver variability was evaluated using Cohen´s kappa with 95 % confidence intervals (CI). The kappa values were computed for each pattern separately with the binary classes “pattern existent” vs. “pattern non-existent” in an examination.

Interlobular septations, intralobular lines and the combination septations and lines were analyzed separately and grouped as “reticular pattern”. Consolidations with air bronchogram and consolidations without air bronchogram were also analyzed grouped as “consolidation”.

The kappa values were graded using the classification proposed by Landis and Koch [18]; k<0.00, no agreement; 0.00 < k ≤ 0.20, slight; 0.21 < k ≤ 0.40, fair; 0.41 < k ≤ 0.60, moderate; 0.61 < k ≤ 0.80, substantial; 0.81 < k ≤ 1.00, near perfect.

The CI of the kappa values regarding the 2011 and 2018 UIP criteria were compared for overlaps. Non-overlapping 95 % CI was considered as statistically significant differences at p = 0.05 level. For each reader, the contingency table of HRCT classified as UIP pattern using 2011 and 2018 criteria was analyzed with McNemar test.

## 3 Results

### 3.1 Interobserver variation in pattern assessment

In the first reading, two observers independently evaluated the examinations and noted all identifiable patterns in each examination. The interobserver variability for the different patterns are shown in Table 1.

**Table 1:** Interobserver variability for parenchymal patterns.

Pattern	n*	Agreement**	Kappa (95 % CI)	Kappa class
**Nodular pattern**				
Perilymphatic nodules	9.5	123/126 (98 %)	0.83 (0.64–1.02)	Near perfect
Tree-in-bud nodules	15.0	122/126 (97 %)	0.85 (0.70-0.99)	Near perfect
Non-tree-in-bud nodules	6.5	119/126 (94 %)	0.44 (0.04-0.84)	Moderate

**Ground glass**	28.5	101/126 (80 %)	0.44 (0.24-0.64)	Moderate

**Reticular pattern**	57.5	107/126 (85 %)	0.70 (0.57-0.82)	Substantial
Interlobular septations	9.5	121/126 (96 %)	0.72 (0.47-0.96)	Substantial
Intralobular lines	19.5	101/126 (80 %)	0.28 (0.03-0.53)	Fair
Septations + lines	43.0	104/126 (83 %)	0.61 (0.46-0.76)	Substantial

**Crazy paving**	13.5	113/126 (90 %)	0.47 (0.19-0.74)	Moderate

**Consolidation**	43.0	110/126 (87 %)	0.72 (0.59-0.85)	Substantial
Without air bronchogram	27.5	107/126 (85 %)	0.56 (0.38-0.74)	Moderate
With air bronchogram	19.5	119/126 (94 %)	0.79 (0.64-0.94)	Substantial

**Decreased attenuation**				
Emphysema	24.0	110/126 (87 %)	0.61 (0.44-0.79)	Substantial
Honey combing	27.0	118/126 (94 %)	0.81 (0.69-0.94)	Near perfect
Other cystic patterns	6.0	124/126 (98 %)	0.83 (0.59–1.07)	Near perfect
**Normal**	16.0	124/126 (98 %)	0.93 (0.83–1.03)	Near perfect

Note: * Average number of cases are the sum of the two observers findings divided by two. ** The number of HRCTs, out of the total 126, in which the two observers agreed on whether a pattern was present or not.

There was a near perfect agreement as to whether the examination was normal or contained one or more patterns, kappa 0.93. For the different parnchymal patterns, there was a large variation in interobserver agreement. Tree-in-bud nodules, perilymphatic nodules, honeycombing and other cystic patterns showed near perfect agreement, while intralobular lines showed the lowest interobserver agreement.

### 3.2 Interobserver variation in 2011 and 2018 UIP criteria

In addition to the identifiable parenchymal patterns, the observers also evaluated whether the findings met the criteria for “UIP Pattern” according to the 2011 UIP criteria in the first reading. In the second reading, the HRCTs were classified according to the 2018 UIP criteria in the four categories “UIP”, “Probable UIP”, “Indeterminate for UIP“ and “Alternative diagnosis”.

The kappa value for the four-class interobserver agreement in the 2018 UIP criteria between reader 1 and 2 was 0.62, substantial agreement. The confusion matrix for the four-class classification is shown in Table 2.

**Table 2:** Confusion matrix 2018 UIP criteria.

Reader 2					
		UIP	Probable UIP	Indeterminate for UIP	Alternative diagnosis
Reader 1	UIP	12	0	1	1
	Probable UIP	1	2	2	0
	Indeterminate for UIP	5	1	5	6
	Alternative diagnosis	1	1	2	86

Note: UIP – usual interstitial pneumonia.

The kappa values using the 2011 and 2018 UIP criteria were similar, see Table 3. In the 2018 criteria assessment, dichotomization at two different levels did not reveal any significant differences in the agreement.

**Table 3:** Inter-reader variation in UIP assessment.

Criteria and dichotomization	Kappa (95 % CI)
2011 criteria (UIP Pattern vs. Possible UIP or Inconsistent with UIP)	0.58 (0.32-0.83)^a^
2018 criteria (UIP Pattern vs. probable UIP, indeterminate for UIP or alternative diagnosis)	0.69 (0.49-0.88)^a^
2018 criteria (UIP pattern or probable UIP vs. indeterminate for UIP or alternative diagnosis)	0.66 (0.47-0.84)^a^

Note: UIP – usual interstitial pneumonia. CI – confidence interval.

a All confidence intervals overlap indicating no statistically significant differences (p > 0.05).

The 95 % confidence intervals of the kappa values overlapped. The null hypothesis of equal kappa values using the 2011 and 2018 UIP criteria could not be rejected – there was no statistically significant difference in agreement using the 2011 and 2018 criteria.

### 3.3 Consistency between 2011 and 2018 UIP criteria

Reader 1 classified nine scans as “UIP Pattern” using the 2011 criteria and 14 scans as “UIP” using the 2018 criteria. Reader 2 classified 17 scans as “UIP Pattern” using the 2011 criteria and 19 scans as “UIP” using the 2018 criteria. The confusion matrices for reader 1 and 2 are shown in Tables 4a, 4b. Using McNemar test, there were no statistically significant differences in the number of HRCT classified as UIP, neither for reader 1 (p = 0.13), nor reader 2 (p = 0.73).

**Table 4a:** Confusion matrix 2011 vs. 2018 UIP criteria, reader 1.

	2011 UIP pattern	2011 Possible UIP or Inconsistent with UIP pattern
2018 UIP	8	6
2018 Probable UIP, Indeterminate for UIP or Alternative diagnosis	1	111

**Table 4b:** Confusion matrix 2011 vs. 2018 UIP criteria, reader 2.

	2011 UIP pattern	2011 Possible UIP or Inconsistent with UIP pattern
2018 UIP	14	5
2018 Probable UIP, Indeterminate for UIP or Alternative diagnosis	3	104

Note: UIP – usual interstitial pneumonia.

## 4 Discussion

In the present study, the interobserver variation in HRCT reading was quantified for most commonly encountered parenchymal patterns, and for the UIP criteria published 2011 and 2018. To the best of our knowledge, this is the first study that quantifies the substantial variation in interobserver agreement between the different patterns.

The interobserver agreement for the different patterns reached from fair to near perfect. Tree-in-bud nodules, perilymphatic nodules and honeycombing showed near perfect agreement, which suggests that these patterns are more easily identified. Lung diseases that predominantly show these patterns, for example bronchiolitis and sarcoidosis [19,20], might therefore have a better interobserver agreement than other lung diseases.

The lowest interobserver agreement is seen for intralobular lines as an isolated finding (kappa 0.28). A possible explanation is the difficulty in distinguishing between subtle fibrotic changes and normal hypoventilation when only supine images are used. The agreement for reticular pattern including intralobular lines was considerable higher.

Kappa values for interobserver agreement cannot be directly compared between different studies using different cohorts. However, the interobserver agreement in the present study is similar to kappa values found in several previous studies. For example, previous studies have shown kappa values for honeycombing between 0.37 and 0.84, compared to 0.81 in the present study [16,21].

Of particular interest is the interreader variability in the application of the criteria for UIP according to the ATS/ERS/JRS/ALAT guidelines. In the present study, there was no significant difference between the agreement using the 2011 criteria and the 2018 update. The kappa value of 0.58 for the 2011 UIP criteria in the present study is also comparable to the results in several other reports. In a large study, Walsh et al. found interobserver variability with kappa values between 0.36 and 0.41 for the same binary score as in the present study, and between 0.45 and 0.51 for weighted kappa, including the class “Possible UIP” [10]. In contrast, a near perfect interreader agreement (kappa 0.92) was seen in one study [21].

The presence of honeycombing in HRCT is a necessary, but not sufficient, condition for UIP Pattern; the distribution should have subpleural and basal predominance, and findings suggestive of another diagnosis should not be present [6,9]. An interesting finding in the present study was that, although the agreement for honeycombing was near perfect (kappa 0.81), the agreement for the UIP criteria was lower, fair-moderate (kappa 0.58-0.69), see Table 3. This finding suggests that the interobserver variations in the UIP criteria to a large degree relate to the distribution of the honeycombing within the lungs and to signs suggestive of another diagnosis.

The present study underlines that, although diagnostic criteria are clearly stated, the application of these criteria remains an area for subjective image interpretation. An additional challenge is that, besides the 2018 ATS/ERS/JRS/ALAT update [6], the Fleischner Society published a white paper on IPF diagnosis the same year [9]. Although to a large degree the same, the wordings for the diagnostic categories are not identical, and the different wordings may result in slightly different interpretations. Even more importantly for the clinical management, the two reviews have different conclusions regarding the need for surgical lung biopsy for patients whose HRCT demonstrate “Probable UIP” pattern [6,9].

It is always necessary to correlate the imaging findings with the clinical findings. Considering the interobserver variations in the present and previous studies, this is especially true in IPF. In IPF, the multi-disciplinary collaboration including radiologists is therefore essential for correct management of the patients [4,6,9].

The inclusion of two readers from the same institution is both a limitation and an advantage in the present study. More readers would have improved the generalizability. On the other hand, it is an advantage that both readers were thoracic radiologists used to reporting HRCT clinically using the same nomenclature. Thereby, the differences in observed kappa values between the patterns are more likely to be caused by inherent characteristics in the patterns, than by local interpretations of the definition of terms.

With the exception of UIP, we only evaluated patterns and not the diagnostic interpretation of the HRCTs in the study. From a clinical point of view, the diagnosis is obviously more important than the pattern. However, since pattern description is the first step in the interpretation of HRCT, the interobserver variation in the pattern description remains important. The uneven distribution of parenchymal patterns in HRCT in clinical context, necessitated a separate HRCT databank, created by one of the readers, with selected cases for the evaluation of the interreader variation. The reader variations in clinical reading might be larger, since unclear cases might not be included in the databank.

In conclusion, there are relatively large interobserver variations in the HRCT assessment for certain patterns and for the 2011 and 2018 UIP criteria. The interreader variations are important to keep in mind especially when there is discordance between the clinical context and the HRCT report.

## Declaration of Competing Interest

The authors declare that there is no conflict of interest.
